# A New Method of Mixed Gas Identification Based on a Convolutional Neural Network for Time Series Classification

**DOI:** 10.3390/s19091960

**Published:** 2019-04-26

**Authors:** Lu Han, Chongchong Yu, Kaitai Xiao, Xia Zhao

**Affiliations:** 1 School of Computer and Information Engineering, Beijing Technology and Business University, Beijing 100048, China; hanlm68@163.com (L.H.); zhaox@btbu.edu.cn (X.Z.); 2 Shenyang Research Institute of China Coal Technology and Engineering Group, Fushun 113122, China; lnfsxiaokt@139.com; 3 State Key Laboratory of Coal Mine Safety Technology, Shenyang Branch of China Coal Research Institute, Shenyang 110016, China

**Keywords:** MOX gas sensors, mixed gas identification, convolutional neural networks, time series classification, analogous-image matrix data

## Abstract

This paper proposes a new method of mixed gas identification based on a convolutional neural network for time series classification. In view of the superiority of convolutional neural networks in the field of computer vision, we applied the concept to the classification of five mixed gas time series data collected by an array of eight MOX gas sensors. Existing convolutional neural networks are mostly used for processing visual data, and are rarely used in gas data classification and have great limitations. Therefore, the idea of mapping time series data into an analogous-image matrix data is proposed. Then, five kinds of convolutional neural networks—VGG-16, VGG-19, ResNet18, ResNet34 and ResNet50—were used to classify and compare five kinds of mixed gases. By adjusting the parameters of the convolutional neural networks, the final gas recognition rate is 96.67%. The experimental results show that the method can classify the gas data quickly and effectively, and effectively combine the gas time series data with classical convolutional neural networks, which provides a new idea for the identification of mixed gases.

## 1. Introduction

The electronic nose is an electronic system that uses the response pattern of a gas sensor array to identify gases. The electronic nose is mainly composed of a gas sensor array, signal preprocessing and pattern recognition, the core of which is the gas sensor array. Gas sensors can be classified into metal oxide type, electrochemical type, conductive polymer type and so on. Currently the most widely used are metal oxide gas sensors called MOX gas sensors [1]. MOX gas sensors have the advantages of small volume, fast response speed, low cost and long service life. Therefore, they are widely used in the field of detection of gases such as industrial exhaust gases, flammable and explosive gases, and the analysis of smells in terms of the intensity of the smell or hedonic quality, etc. [2,3,4,5,6]. MOX gas sensors cause a change in resistance by physicochemical reaction with the gas to be measured, and convert information about gas type and concentration into a single signal output [7]. The sensor array can obtain multi-path response signals, which provides a feasible means for detecting and analyzing the composition of mixed gases. The types and number of sensors used is determined by the nature of the gas being measured [8,9].

For the identification of mixed gas components, the pattern recognition method largely determines the recognition accuracy. Therefore, the mixed gas component can be effectively detected by an improved identification method. The existing methods for identification of mixed gases are roughly divided into three types: (1) Gas chromatography-mass spectrometry (GC-MS) method; it utilizes the physical properties of multi-component gases to identify them with high sensitivity and high separation efficiency, and any gas mixture having high reproducibility can be accurately identified both quantitatively and qualitatively, but the technique has limitations due to the expensive apparatus needed, the huge time required for the analysis, and the need for a specialized operator [10]. (2) Data-driven approach. It is a method of applying technical methods such as statistics or machine learning for classification [11]. The structure of shallow neural networks is relatively simple, and is generally determined by empirical methods, which will cause the problem of gas recognition accuracy to be reduced to some extent. The number of support vectors in SVM increases linearly as the number of training samples increases, the sparsity of the model will be greatly reduced, the parameters need to be optimized to achieve the best recognition rate, and the process of parameter optimization greatly increases the amount of calculation. (3) Fusion method. A variety of statistical or machine learning methods are combined to classify signals, which can improve the classification accuracy to a certain extent [12]. Table 1 summarizes the advantages and disadvantages of the three methods for identifying components in a gas mixture. However, the gas time series data has complex features, large dimensions, and data implicit patterns are difficult to mine. The above algorithms are computationally intensive, and the ideal effect cannot be achieved in a big data environment.

In recent years, with the continuous development of deep learning technology, some deep learning models have gradually been applied to the study of classification problems [13,14,15,16]. The deep learning model is a deep neural network model with multiple nonlinear mapping levels, which can abstract the input signal layer by layer and extract features to dig deeper potential laws. Among many deep learning models, convolutional neural networks have good effects in image classification and other applications, but they are limited in the field of gas classification [17,18,19]. Reference [20] first proposed the idea of using the deep convolutional neural network (DCNN) for gas classification and designed a neural network called GasNet. Reference [21] uses a simpler LeNet-5 network for gas classification. Reference [22] presents a novel one-dimensional deep convolutional neural network (1D-DCNN) for automatically extracting features. These deep learning models applied to classify gas data have a simple structure, a small amount of input data, and directly operate on the acquired two-dimensional time series data. However, due to the limitation of input data, some models such as VGG and Google-Net for image classification cannot be directly applied to classified mixed gas data. 

Therefore, this paper will design a method to classify mixed gas by using the existing convolutional neural networks (CNN) model. That is, mapping the original gas time series data into an analogous-image matrix and using the existing classical CNN model to extract features. Then, the purpose of classifying the mixed gas is achieved. The proposed method can not only overcome the problem that the traditional classification method does not accurately identify the time series data category pattern, but also can use the convolution operation of CNN to extract more comprehensive features of matrix data, and provide a new idea for sensor mixed gas classification. The contributions of this paper can be summarized as follows:

(1) Mapping the gas raw data from the time series data to the analogous-image matrix, and transforming the mapping manner to form four sample sets 1 to 4 for experiment;
(2) Using the Visual Geometry Group-16 (VGG-16), Visual Geometry Group-19 (VGG-19), Residual Network 18 (ResNet18), Residual Network 34 (ResNet34) and Residual Network 50 (ResNet50) in the CNN models to classify the analogous-image matrix data. The accuracy of the proposed method was verified by five categories of CO, methane and ethylene mixed gases with different concentrations of the different components.

This paper is organized as follows: Section 2 analyzes the existing deep learning methods for mixed gas classification and describes the overall procedure of mixed gas classification. Section 3 introduced the original data set and the specific steps of three mapping methods of mapping the time series data to the analogous-image matrix. Section 4 introduces the model principle and characteristics of convolutional neural networks and recurrent neural network, and Section 5 presents performance evaluation results that verify the superiority of the proposed method. Finally, Section 6 describes the conclusions of this study and our future research plans.

## 2. Related Works

Regarding the study of mixed gas classification, the most commonly used research methods are machine learning methods. For example, Krivetskiy used a random forest, support vector machine and shallow multi-layer perceptron algorithm to selectively detect the presence of low concentrations of individual gases [23], Fonollosa proposed the so-called inhibitory support vector machines method to identify whether ethylene is present in a mixed gas [24]. The idea of classifying mixed gases using deep convolutional neural networks was proposed by Pai [20], who applied the “deep” learning model to gas classification for the first time. Based on this idea, Wei used the existing LetNet-5 model which applied to the handwritten dataset to classify the mixed gas [21]. Zhao present a novel one-dimensional deep convolutional neural network (1D-DCNN) for comprehensively and automatically extracting features and classifying mixture gases [22].

In [20], a deep convolutional neural network called GasNet was proposed for gas classification. The network has 38 layers, including six convolution blocks, global average pooling layer, and a fully connected layer. Each of the convolution blocks consists of six layers, including two convolutional layers, two bulk normalization layers, and two rectification linear unit (ReLU) layers to extract representative features. The classification results are obtained using a fully connected layer with multiple types of neurons and Softmax activation function.

In [21], a new LeNet-5 gas recognition convolutional neural network structure for electronic noses was proposed. LeNet-5 is a typical and widely adopted model that has been proposed by the predecessors. The authors apply this network to the field of gas identification and reduce convolutions to speed up calculations. The original data size is 480 × 12, the input data is extracted every 40 points of the sensor samples, and the final input data size is 12 × 12. The output layer contains three neurons based on the target, corresponding to three target categories.

In [21,22], a novel one-dimensional deep convolutional neural network (1D-DCNN) was proposed. The original input data (16 × 100) was expanded into a one-dimensional vector, so the new input data is a one-dimensional vector with size of 1 × 1600. And the feature was extracted by using a one-dimensional filter (1 × 16 × 8, 1 × 3 × 64, 1 × 3 × 64 and 1 × 3 × 128). The 1D-DCNN with multi-label way not only significantly reduces the label dimension but also quantifies the probability of each component in mixed gases.

The above convolution deep neural networks for gas recognition are basic models, and there are the following problems in application. First, the original data size is m × n, where m is the number of sampling points, and n is the number of sensors. Generally, m is much larger than n, which cannot be directly used as input data, and m-dimensional data needs to be extracted. The extracted data will lose a lot of valid information, which is easy to produce large errors. Second, such input data cannot use the CNN model with more complex network structure.

In this paper, in view of the above problems, we propose a new method for mapping the raw data into analogous-image matrix data and then using the advantages of convolutional neural networks in image classification for gas recognition. First, the deep learning model is quite different from the traditional machine learning approach. Traditional machine learning methods assume that sample feature representations are given and that specific machine learning algorithms are designed. However, the important idea of deep learning is “end-to-end” learning, that is, there is no need to think about sub-problems in the whole learning process. Furthermore, when mapping the original time series data into analogous-image matrix data, it is not necessary to reduce the data dimension, and the feature information of the data can be retained to the greatest extent without considering the complexity of the model. In addition, the convolution operation of the CNN model can be used to extract more comprehensive feature information in the data and improve the classification accuracy.

**Figure 1:**
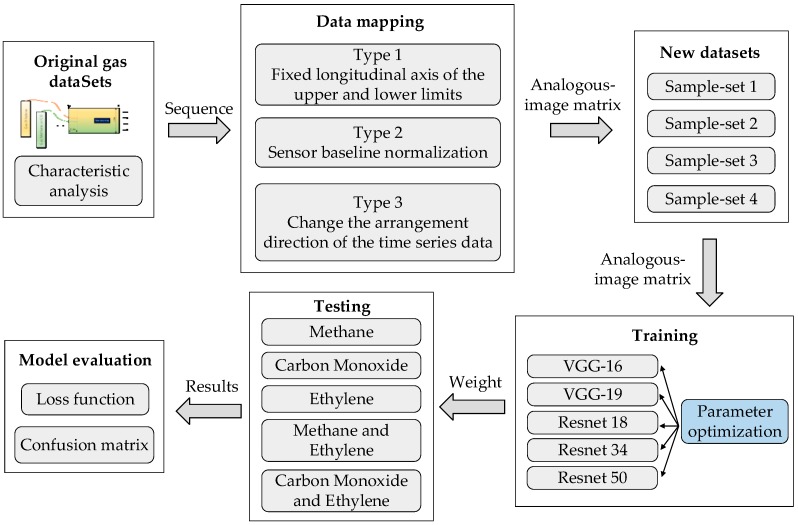
Overall processing procedure.

The overall processing procedure of the proposed method is shown in Figure 1. First, the original time series data set is introduced in “Original gas datasets”, and the nonlinearity of the data is analyzed. Then three data mapping methods are proposed in “Data mapping”, and mapping the sequence data into an analogous-image matrix. “New Datasets” are four new sample sets after data mapping, which used as experimental data and divided training and test samples. The training sets are trained with five kinds of Convolutional Neural Networks including VGG-16, VGG-19, Resnet18, Resnet34 and Resnet50 in “Training”. The test sets are classified in “Testing”, and finally the model is evaluated with loss function and confusion matrix in the “Model evaluation”. 

## 3. Data Processing

### 3.1. Original Gas Datasets

The experimental data is based on the UCI public data set "Gas sensor array exposed to turbulent gas mixtures data set". The experimental device for data acquisition was a 2.5 m × 1.2 m × 0.4 m wind tunnel facility with two gas sources (labeled as gas source 1 and gas source 2). Each gas source is independently controlled to release gas at different flows, gas source 1 releases ethylene, and gas source 2 releases methane or CO. Thus, a mixture of “methane and ethylene” or “CO and ethylene” is formed. The sensor array is placed in a wind tunnel where the wind turbine generates a gas stream that is naturally mixed along the air stream to produce a gas mixture of different concentration levels at the location of the sensor array. And constantly replace the mixed gas to the exhaust port [24].

**Figure 2:**
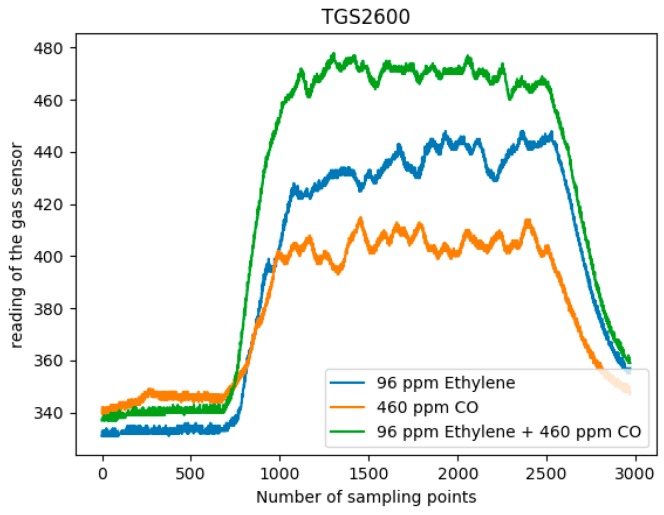
TGS2600 sensor response process curve for single and mixed gases.

Among them, the sensor array consists of eight commercial MOX gas sensors (Figaro USA Inc., Glenview, IL, USA) of six types. The sensor information of the sensor array is shown in Table 2 [24]. Among them, there are two TGS2602 andTGS2620 sensors, and one of the other types of sensor. The target gases and sensitivity of the sensors are different. The TGS2602 has high sensitivity to low concentration gases, and TGS2620 can detect methane and ethylene at the same time with different sensitivity. Because of the different locations of the sensors, the response values of the same sensors also vary. Table 3 is a sample of the original data (high concentration of ethylene and zero concentration of CO), and the data before 3 s is omitted. These MOX gas sensors rely on changes in the resistance of a semiconductor oxide film to react with gas molecules to detect gases. Therefore, when two gas sources release reducing gases (ethylene, methane and CO), the sensor response will be different. Moreover, the sensor response has a nonlinear characteristic in single and mixed gases. That is, the response output of the sensor to the mixed gas is not equal to the sum of the responses of the sensor to the two target gases respectively. Therefore, for the characteristics of sensitive materials of MOX gas sensors, it is not possible to obtain the exact information of the target gas type and concentration through the output of the sensor. It is necessary to cooperate with the signal processing method to reveal the information of the target gas contained in the response signal. Figure 2 shows the response curve of the TGS2600 sensor for single ethylene, CO and a mixture of the two samples.

These MOX gas sensors produce a time-dependent multivariate response to different gases. The operating temperature of the sensor is controlled by a built-in heater that maintains a constant voltage of 5 V. The inspection platform also includes temperature and relative humidity sensors. 

The concentration of gas released at the two sources is constant, 2500 ppm for ethylene, 1000 ppm for methane and 4000 ppm for CO, respectively. Under the action of the wind current generated by the wind turbine, the gas released by the gas source flows at different flow rates, causing the gas concentration to be diluted to varying degrees. When the gas reaches the sensor, it mixes with each other at different concentrations. Finally, the average concentration levels measured at the sensor array locations for three gases at four different wind speeds (zero, low, medium and high) are provided, as shown in Table 4.

Based on the gas concentrations shown in Table 4, a total of 30 kinds of the mixture configuration with different concentrations were formed: 15 kinds of a mixture of CO and ethylene mixtures, 15 kinds of a mixture of ethylene and methane. Each configuration was repeated six times for a total of 180 sets of raw data. For each mixed configuration, when the concentration of one is zero, it becomes a single gas. Therefore, the classification aims to identify five gases, namely pure CO, pure methane, pure ethylene, CO-ethylene, methane-ethylene. Each measurement lasts for 300 s in the following manner: initially no gas is released and clean air flows along the wind tunnel. At 60 s, both gas sources begin to release the corresponding gas at the specified flow rate for 180 s. Finally, the system achieved a 60 s baseline recovery. The signal of one sensor is acquired every 100 ms, so a sequence of 3000 is acquired for 300 s. Eight sensors produce eight sequence, so each sample size is 3000 × 8. 

### 3.2. Data Mapping

The raw data A is a two-dimensional sequence matrix of size m × n, as in Equation (1), where m = 2970 is the number of sampling points (the values of the first three seconds are omitted), n = 8 is the number of sensors, and element *a_ij_* in A is the response value of the *j*-th sensor at time *t*. In addition, a time factor is added to the general two-dimensional matrix, such as the formula (2). That is, *a_t+_*_1,*j*_ is the value of the next moment of *a_ij_*, and the order of the two cannot be changed. For such a matrix, m and n have a relationship as shown in Equation (3), and the difference in the size of m and n causes a serious imbalance between the rows and columns of the matrix. When the feature extraction is performed on the original data by using the deep learning algorithm, the m-row data needs to be reduced, which will inevitably lead to the loss of effective information. If the original data is completely preserved, the application of the classical CNN models will be limited. Therefore, this paper proposes to map the original time series data into analogous-image matrix data, and extract more comprehensive features in the data through the convolution operation of CNN, thereby improving the accuracy. Specific steps are as follows:

(1) $$A={\left[\begin{array}{cccc} a_{11} & a_{12} & \cdots & a_{1n}\\ a_{21} & a_{22} & \cdots & a_{2n}\\ a_{31} & a_{32} & \cdots & a_{3n}\\ \cdots & \cdots & & \cdots \\ a_{m1} & a_{m2} & \cdots & a_{mn} \end{array}\right]}_{2970\times 8}$$

(2) $$(a_{1,j},a_{2,j},\ldots ,a_{t-1,j},a_{t,j},a_{t+1,j},\ldots ,a_{2970,j})\;$$

*m* >> *n* (3)

*Step 1*. The response value of the sensor array is mapped to the analogous-image matrix which is converted from 2970 × 8 to 640 × 480. The mapping process is as follows: First, designing an empty matrix B with size of 640 × 480, and dividing the horizontal axis into 2970 parts. Then extracting the maximum value M in the original data, dividing the vertical axis into M parts. Second, fill the element *a_ij_* in A to the *b_t(aij)_* position of the empty matrix. That is, mapped to a new two-dimensional matrix B_1_. Third, normalizing the elements with values in B_1_ to 0~255, which represents the gray level in the analogous-image matrix. Finally, the elements with no values in the matrix B_1_ are set to 255. 

**Figure 3:**
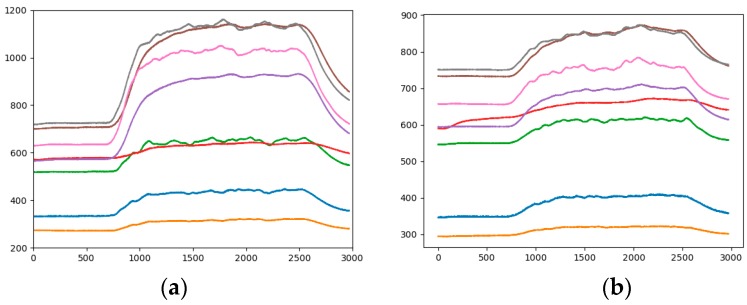
A sample of analogous-image (**a**) High concentration of Methane; (**b**) Low concentration of Ethylene.

**Figure 4:**
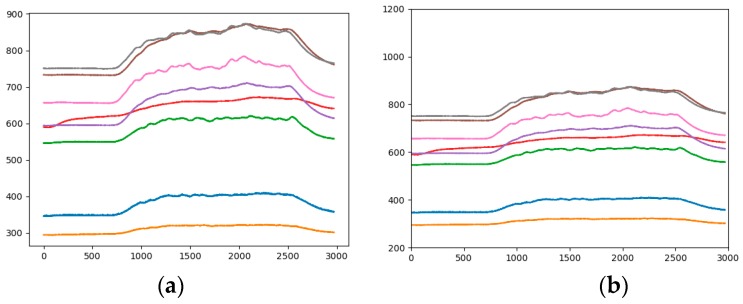
Analogous-image of low concentration of Ethylene (**a**) Unfixed range of response values; (**b**) Fixed range of response values.

**Figure 5:**
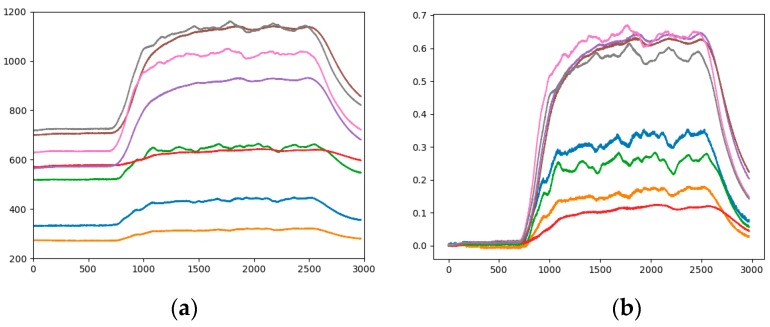
Analogous-image of high concentration of Ethylene (**a**) The raw data; (**b**) The standardized data.

**Figure 6:**
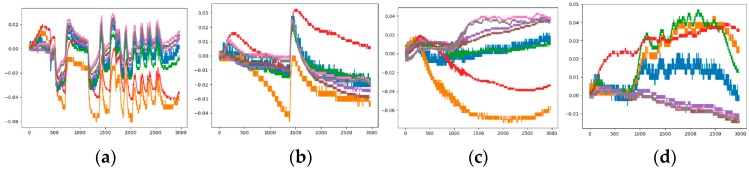
Unusual analogous-image (**a**) Low concentration of Methane; (**b**) Low concentration of Methane and Low concentration of Ethylene; (**c**) Low concentration of Methane and Medium concentration of Ethylene; (**d**) Low concentration of Methane.

**Figure 7:**
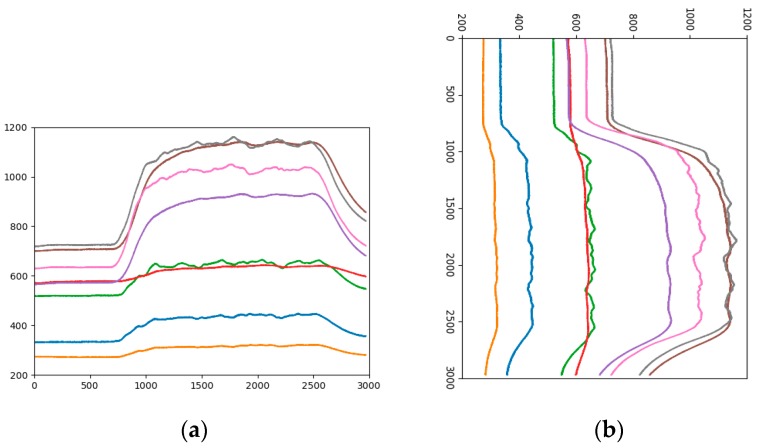
Analogous-image of high concentration of Ethylene (**a**) Horizontal arrangement, (**b**) Vertical arrangement.

*Step 2*. In order to highlight the difference of different sensors, the gray image is converted into RGBA image with size of 640 × 480 × 4. The RGBA image is converted into a RGB image with different size for different networks. The time series data of eight sensor arrays are displayed in one analogous-image and represented by different colors. As shown in Figure 3, in different analogous-image, the data of the same sensor is represented by the same color. The numerical value of the original data reflects the position in the analogous-image. The relative position of the large value is on the upper side, and the relative position of the small value is lower (the horizontal axis of Figure 3, Figure 4, Figure 5, Figure 6 and Figure 7 represent the seconds that be multiplied by 10, that is number of sampling points. The vertical axis of Figure 3, Figure 4, Figure 5, Figure 6 and Figure 7 represents the response value of the sensor).

It can be seen from Figure 3 that 0 to 60 s is the "preparation time" of the sensor. At this stage, air is input and the data is smoothly changed. We start to release the mixture at 60 s and this lasts for 180 s, the two gas sources began to release the corresponding gas, and the sensor response value is gradually increased. The magnitude and speed of the increased vary depending on the type of sensor. Moreover, the law of fluctuations is also different. This period is the "response time" of the sensor. The gas is stopped for 240 s to 300 s, which is the "recovery time" of the sensor, and the sensor returns to the baseline value.

*Step 3.* Change the mapping method of the original data, such as changing the relative position of the original value in the analogous-image, unifying the sensor baseline and changing the direction of the data develops in the analogous-image. Different sample sets were formed by different mapping methods for comparison experiments.

#### 3.2.1. Fixed Range of Response Values

The range of sensor response values is different, such as in Figure 3a it is 200 to 1200, and in Figure 3b it is 300 to 900. Taking the gray curve as an example, the values are different but the height positions of the curves in the picture are very similar. The effect of the sensor response value on the category is not well reflected, and the feature gap between the categories is reduced. Therefore, the impact of response values on the height of the analogous-image needs to be considered in data mapping. By statistics, all sensor response values are between 200 and 1200, so the range of sensor response values of all samples is fixed at 200 to 1200. Figure 4b is the analogous-image after fixing the response values range of Figure 4a. We set the sample set of the fixed range as Sample-set 2.

It can be seen from the comparison between Figure 4a,b that the position of each curve has changed in the image, but the relative positions of the curves is constant. The effect of the sensor response value on the position of the curve in the image is shown between different pictures. The color of the curve remains the same, eliminating the influence of the curve color on the classification result.

#### 3.2.2. Sensor Baseline Standardization

The difference in baseline values for different sensors is slightly larger. In order to eliminate the impact of the baseline on the data and ensure the reliability of the data, the general pattern recognition algorithm first take the standardized operation to the data [12]. The method is as shown in Equation (4), where X_Standard value_ is the value obtained after subtracting the baseline treatment, which we call: “standard value”, X_Response value_ is the true value of the sensor during the response, X_Baseline value_ is the baseline value of the sensor in air or standard gas. That is, the “Standard value” is equal to the difference between “Response value” and the “Baseline value” divided by the “Baseline value”. The “Standard value” data can effectively eliminate the impact of the environment and minimize environmental errors:

(4) $$X_{\mathrm{Standard}\;\mathrm{value}}=\frac{X_{\mathrm{Response}\;\mathrm{value}}{\;-\;X}_{\mathrm{Baseline}\;\mathrm{value}}}{X_{\mathrm{Baseline}\;\mathrm{value}}}$$

Since this experiment adopts the convolutional neural network analysis method, and does not directly classify the sensor data, it is necessary to explore whether the “Baseline value” has an impact on the experimental results. Table 5 is the standardized data of Table 3. 

Figure 5 is a comparison chart before and after standardization. It is observed from the figure that the basic trend of the curve has not changed, but the relative position of each curve has changed. For example, the positions of the yellow and blue curves are relatively elevated, while the position of the red curve is decreased. This change will cause a corresponding change in the characteristics of the category to which the image belongs. 

After the standardization operation, the observed sample set have four unusual images as shown in Figure 6, which do not meet the “preparation time”, “response time” and “recovery time” that a normal sample should have. The entire response process is cluttered. This may be caused by environment factor, possible damage or non-calibration of the sensor itself. If the unusual image is placed in the training sample, it will affect the effect of classification, so they need to be eliminated. The sample set after normalization and denoising is the Sample-set 3.

#### 3.2.3. Changing the Arranged Direction of Data

The biggest difference between the time series data and the general visual data is that the time series data have a time label, while the general picture does not contain the time information and changing the arrangement direction of data will not affect the shape of the object in the image, but for time series data, a change the direction of data development can lead to large changes in curve trends and locations. Therefore, when classifying analogous-image matrices by CNN, it is necessary to consider the influence of the arranged direction of data on the experimental results. Figure 7a is an analogous-image in which the data is arranged horizontally, the horizontal axis is the number of data points, and the vertical axis is the sensor response value. The size of the analogous-image is 640 × 480 × 3. Figure 7b is an analogous-image in which the data is vertically arranged. Currently, the horizontal axis is the sensor response value, the vertical axis is the number of data points. In order to conform to the order of the convolution operation, the time -series of the vertical axis is set from top to bottom. The size of the analogous-image becomes 480 × 640 × 3. Let the sample set which the data is vertically arranged be the Sample-set 4.

The above are three ways to map time series data into class picture matrix, which considering the characteristics of the sensor and the feature of time series data may affect the classification result. Three data sets were created in three ways to find the most appropriate mapping method.

## 4. Gas Classification Method in Dynamic Mixtures

Convolutional neural networks are a special type of artificial neural network, which are different from other neural network models, such as recurrent neural networks, Boltzmann machines, etc. Their main feature is a convolution operation. Therefore, CNN perform well in the fields of image classification and image segmentation. This section briefly introduces the structure of the CNN model, including the input layer, output layer and hidden layer, as well as the typical architecture VGG, Resnet of the CNN.

### 4.1. CNN Structure

**Figure 8:**
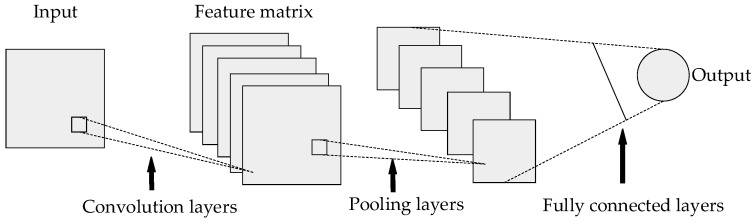
CNN overall structure.

CNN is a hierarchical model consisting of input and output layers and multiple hidden layers [25,26,27]. Inputs are RGB images, audio data, and so on. CNN extracts high-level semantic information from input data through layer-by-layer stacking of convolution, pooling and nonlinear activation function mapping, and abstracts it layer by layer. This process is called “feedforward operation”. Among them, different types of operations are generally referred to as “layers” in convolutional neural networks, convolution operations correspond to “convolution layers”, pooling operations correspond to “pooling layers”, and so on. Finally, the last layer of CNN formalizes the target tasks (classification, regression, etc.) into objective functions. By calculating the error or loss between the predicted value and the true value, the error or loss is fed back from the last layer by the back-propagation algorithm [28]. The parameters of each layer are updated, and the feedforward is fed again after updating the parameters until the network model converges, thus achieving the purpose of model training. The overall structure of CNN is shown in Figure 8.

In the entire CNN structure, the input of each neuron in the convolutional layer is connected to the upper layer, and the local feature extraction is performed. After entering the pooling layer, the commonly used pooling operations have average pooling and maximum pooling. This operation has characteristic invariance, feature dimension reduction, and prevents over-fitting to a certain extent. The convolutional layer and the pooled layer have different numbers in different scenarios. In order to increase the expressiveness (non-linearity) of the entire network, an activation function is added after the pooling layer to map the results of the previous linear operation layer into a nonlinear function. The fully connected layer acts as a “classifier” throughout the CNN. Therefore, for the input graph, various features are first extracted through the convolution layer, then the secondary feature extraction is performed through the pooling layer, and the output of the pooling layer is mapped to more complex nonlinear features through the activation function layer. Finally, the fully connected layer maps the learned feature representations to the label space of the sample for prediction.

Compared with traditional neural networks, CNN has three major differences: local perception, weight sharing and multi-convolution kernel. The traditional neural network adopts the method that the input layer and the hidden layer are fully connected. When the image size is relatively large, the full connection method increases the amount of calculation. Therefore, the CNN adopts a local connection method in the convolutional layer, and obtains local information and local features of the image by applying a convolution kernel or a filter of a certain size to the local image region. The parameters of the convolution kernel are treated as weights, which apply not only to one local input, but to all inputs at different locations. Therefore, weight sharing means that the entire image shares a set of convolution kernel parameters, which can reduce the amount of calculation and improve the calculation efficiency. A convolution kernel extracts a feature. To obtain more different feature sets, the convolutional layer has multiple convolution kernels to extract different features.

### 4.2. CNN Implementation Architecture

#### 4.2.1. VGG-Nets

**Figure 9:**
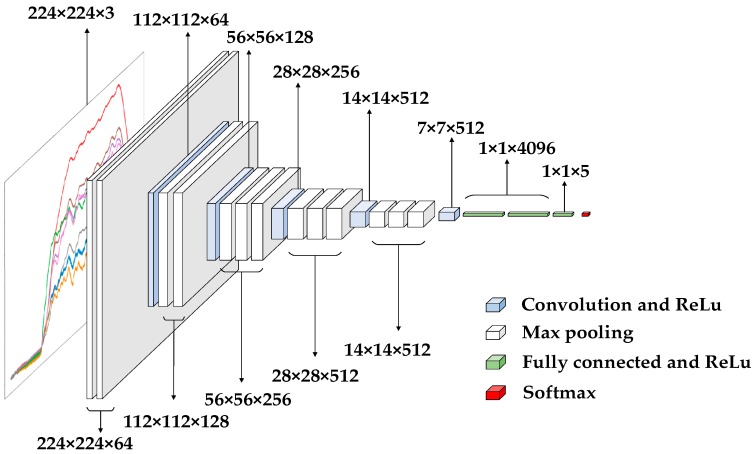
The network architecture of VGG-16.

VGG-Nets [29,30,31] was proposed by the Visual Geometry Group (VGG), a well-known research group at Oxford University in the UK. Due to its good generalization performance, VGG-Nets can improve classification accuracy by using pre-trained model on ImageNet dataset. Typical are VGG-16 and VGG-19, with the difference being the number of convolution layers. Taking the VGG-16 network as an example, the network architecture is shown in Figure 9. The “16” means a 13-layer convolutional layer and a 3-layer fully-connected layer. In order to increase the network depth, and then increase the model capacity and model complexity, small convolution kernels (3*3) are commonly used in VGG-16. And the size of input data of network is 224 × 224 × 3. The ReLU function [32,33] be used as an activation function, which is better than the tanh and sigmoid functions. The function expression such as Equation (5), where x is the independent variable:

(5) ReLU(x)=max{0,x}={x   x≥00   x<0

Compared to other activation functions, ReLU has the following advantages: For linear functions, ReLU is more expressive, especially in deep networks; for nonlinear functions, Since the gradient of ReLU is constant in the non-negative interval, there is no vanishing gradient problem, which keeps the convergence speed of the model in a stable state.

#### 4.2.2. Resnet

**Figure 10:**
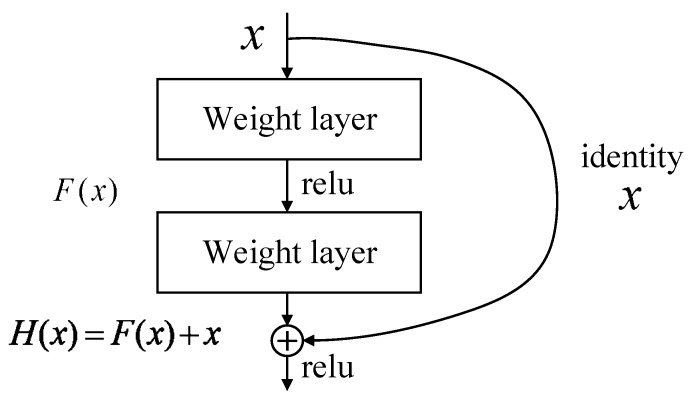
Residual learning module [34].

The depth and width of the neural network are two core factors that characterize the complexity of the network. The depth is more effective than the width in increasing the complexity of the network, but as the depth increases, the training becomes more difficult. This is mainly because in the network training process based on stochastic gradient descent, the multilayer backpropagation of the error signal can easily lead to the gradient “dispersion” or the gradient “disappear”. Moreover, there is a phenomenon that the training error increases as the depth increases [34,35]. In order to solve this problem, a residual network [34] appeared. Because the residual network solves the problem of training difficulty caused by network depth, its network performance (accuracy and precision) far exceeds the traditional network model. Figure 10 shows the residual learning module [34], which has two branches, one is the left residual function F(x) and the other is the identical mapping “x” of input. After the two branches are integrated by the corresponding elements, they are subjected to nonlinear transformation (ReLU activation function) to form the entire residual learning module. A network structure in which a plurality of residual modules is stacked is called “residual network”. Typical residual networks include Resnet18, Resnet34, Resnet50, and so on.

## 5. Experimental Results

Table 6 summarizes the performance evaluation environment. The performance evaluation program was implemented using Python.

### 5.1. Experimental Data

The configuration of 30 mixed concentrations of mixed gases and the number of measurements for each configuration are presented in Section 3.1. The data is distributed in batches, consisting of six measurements per batch, with the same configuration repeated for each measurement. Data is distributed in batches, and each mixed concentration is configured as one batch. Each batch consists of six measurements for a total of 180 measurements, each measurement repeats the same configuration. The 180 measurements data is converted into analogous-image matrix data to form a sample set. In Section 3.2, four sample sets are introduced, namely Sample-set 1, Sample-set 2, Sample-set 3 and Sample-set 4 (Sample-set 1~sample-set 4 will be used to represent the sample set in the following experiments). This experiment divides the 180 measurements in each sample set into five categories, namely: methane, CO, ethylene, CO-ethylene, methane-ethylene. The corresponding labels are 1, 2, 3, 4, 5 (in the following experiments, labels 1-5 will be used to indicate the corresponding category). By analyzing and comparing the test accuracy of different sample sets, the sample set with the highest test accuracy is determined as the most suitable sample set. The gas concentration configuration and total number of samples for each category are shown in Table 7. Where “N” represents 0 concentration, “L, M, and H” represent low, medium, and high concentrations respectively. Therefore, each category consists of a mixture of gases at different concentrations. The accuracy of each classifier was estimated by the ability to correctly classify the five mixed gases using a test set that was not used during training.

The training set and the test set samples were determined by random selection of each concentration of mixed gas at 5:1. The number of samples of the original dataset can be seen in Table 8. For categories 1 and 2, there are 15 samples of training data and three samples of test data. Fewer training samples will affect the training effect. Therefore, we do random micro-float of the original data without changing the characteristics of the data, expand each type of training data to 90 per class, and expand the test data to 27 per class. The number of samples of the extended dataset which is used in subsequent experiments also can be seen in Table 8.

Five kinds of CNN: VGG-16, VGG-19, Resnet18, Resnet34 and Resnet50, were used for comparative experiments, and the best experimental results were achieved by changing the network parameters. We use the accuracy, loss function and confusion matrix to evaluate the performance of the network at the same time. The model parameters corresponding to the experimental model are shown in Table 9. The VGG and Resnet networks have been used to classify ImageNet datasets and obtain weight parameters, which as the pre-training weights can greatly reduce training time. To use the pre-training weights, the input analogous-images of VGG-16 and VGG-19 are 224 × 224 × 3, and the input analogous-images of Resnet18, Resnet34, and Resnet50 are 640 × 480 × 3.

The following sections describe the experimental results of different network models for different sample sets, including two parts of the classification results of different sample sets and the classification results of different network models. The most suitable data mapping method is determined by the experimental results of different sample sets. Then, the classification effect and performance of each model are compared to find the best performing experimental model, which is to find the most suitable feature extraction method for gas data classification.

### 5.2. Datasets Comparison

#### 5.2.1. Sample-set 1 and Sample-set 2

In order to compare whether the stable range of response values influence the experimental results, experiments were carried out using Sample-set 1 and Sample-set 2, and the experimental results are shown in Table 10. For Sample-set 1 with unfixed range of response values, the accuracy of Resnet34 is 100%. Resnet50 increases the convolution layer than Resnet34, but the accuracy decreases with the increase of the number of convolution layers, which may be over-fitting. The minimum accuracy of VGG-16 is 40%, with an average of 73.998%. For Sample-set 2 with fixed range of response values, Resnet50 has the highest accuracy of 96.67%, VGG-19 has the lowest accuracy of 60%, and the average is 80.67%. By comparing the test results of Sample-set 1 and Sample-set 2 of each model, the following conclusions are obtained: although the highest accuracy of Sample-set 2 is slightly lower than that of Sample-set 1, the minimum accuracy of it is much higher, and the average accuracy of Sample-set 2 is significantly higher than that of Sample-set 1. 

The experimental results illustrate the importance of fixed range of response values for the classification results. At the same time, the test accuracy of the Resnet is much higher than that of the VGG, indicating that for such a sample set which with small difference within the class, the Resnet can extract more accurate features and better discrimination.

**Figure 11:**
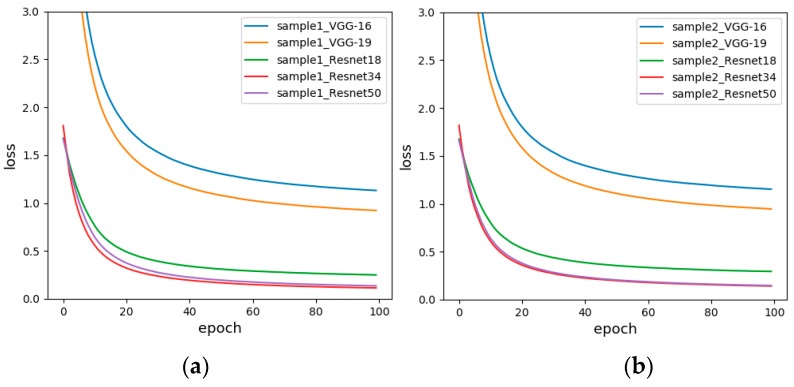
The loss functions before and after fixing range response values, (**a**) Unfixed range of response values (Sample-set 1); (**b**) Fixed range of response values (Sample-set 2).

In order to compare the test results of each model more comprehensively, we draw the loss function of different models for the same sample set. The loss function is used to estimate the degree of inconsistency between the predicted and actual values of the model. It is a non-negative real-valued function, usually represented by an L1 or L2 regular term. The loss function used by our network is the cross-entropy loss function. The loss graph reflects the convergence speed of the model and the proximity to the true value. The faster the convergence and the smaller the loss value, the better the classification effect. Figure 11 shows the loss diagrams of Sample-set 1 and Sample-set 2. It is apparent from both figures that the loss curves of all models gradually decrease and tend to be flat. Moreover, the loss value of the Resnet is much lower than the loss value of the VGG, which is determined by the network structure of the residual network. Residual network solves the problem that the gradient of the VGG-Net disappears when the network depth is deepened. Therefore, the experimental error is greatly reduced. This is consistent with the test accuracy in Table 10. At the same time, in Resnet, Resnet34 and Resnet50 have higher performance than Resnet18, and the convergence speed and minimum value are better than Resnet18. Therefore, the subsequent experiment was performed with fixed range of response values.

#### 5.2.2. Sample-set 2 and Sample-set 3

Based on the experimental results in Section 5.2.1, in order to compare whether the sensor baseline influences the experimental results, the sensor response value corresponding to each sample in Sample-set 2 is normalized, and the resulting value forms new analogous-image matrix with the same size to formed Sample-set 3. The experimental results are shown in Table 11. The results show that the minimum accuracy of Sample-set 3 is 46.67%, the highest is 96.67%, and the test accuracy of each model of Sample-set 2 is greater or equal to Sample-set 3. The experimental results fully demonstrate that the CNN can ignore the impact of the baseline of sensor on the data, making data processing easier. And after standardization, the average recognition accuracy has decreased. It is worth noting that in both sample sets, the test accuracy of VGG-16 is greater than the test accuracy of VGG-19. It might be that as the depth of the network increases, network training is more difficult, and the phenomenon of “disappearance” or “explosion” occurs, which increases the training error.

**Figure 12:**
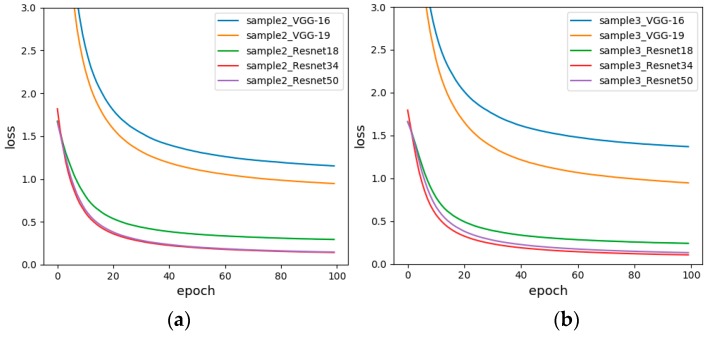
The loss functions before and after data standardization (**a**) Sensor baseline non-standardized (Sample-set 2); (**b**) Sensor baseline standardized (Sample-set 3).

Figure 12 is a loss diagrams of Sample-set 2 and Sample-set 3. It can be seen from the figure that the loss curves of the Resnet of the two graphs are very similar, while the loss curves of the VGG are significantly different. For Sample-set 3, the loss value of VGG16 eventually drops to 1.3683. For Sample-set 2, the loss value of VGG16 eventually drops to 1.1520, and the loss curve of VGG19 converges a little faster. Therefore, a non-standardized approach was chosen for subsequent experiments.

#### 5.2.3. Sample-set 2 and Sample-set 4

Based on the experimental results in Section 5.2.2, in order to compare whether the data arrangement direction influences the experimental results, each sample data in Sample-set 2 is changed to a vertical arrangement to form Sample-set 4. The experimental results are shown in Table 12. For Sample-set 4, Resnet50 has a maximum accuracy of 96.67%, while VGG-16 has a minimum accuracy of 70% and the average is 82.668%, which is better than Sample-set 2.

**Figure 13:**
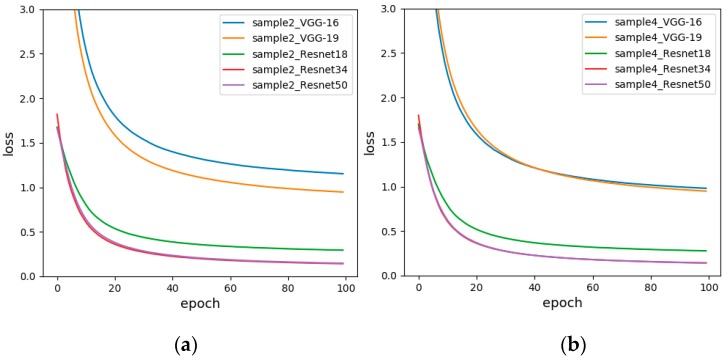
The loss functions of changing the data arrangement direction (**a**) Horizontal arrangement of data (Sample-set 2); (**b**) Vertical arrangement of data (Sample-set 4).

Figure 13 is the loss diagram of Sample-set 2 and Sample-set 4. It is obvious that the convergence speed and minimum value of VGG16 of Sample-set 4 are significantly better than Sample-set 2. For Sample-set 4, the loss function of Resnet34 and Resnet50 basically coincide, but the loss function of Resnet50 converges faster, down to 0.1414 in 100 generations. For Sample-set 2, the loss function of Resnet34 converges the fastest, eventually reaching around 0.1419.

By comparing the experimental results with VGG-16, VGG-19, Resnet18, Resnet34 and Resnet50 of Sample-set 1~4, it is determined that Sample-set 4 is the best sample set, which is, the highest recognition rate is 96.67% and the average recognition rate is up to 82.668% for Sample-set 4. The experimental results show that the analogous-image matrix of gas sensor data mapping has the following characteristics in data processing:

(1) For general image such as ImageNet classification, the position of the target object in the image does not affect the category of the image, but for gas data, position information is an important feature, which increases the difficulty of classification.
(2) When using general pattern recognition classification algorithms such as PCA, SVM, and shallow neural networks for time series data classification, the original sensor data is analyzed, but the baseline of each sensor is different. In order to eliminate the impact of the baseline on the data, the data is first standardized. However, when using the convolutional neural network for feature extraction, this step is not required, which not only simplifies the operation but also improves the accuracy.
(3) Due to the particularity of the time series data, the data has a time stamp. Similarly, the convolution operation of the convolutional neural network is also performed in a certain order. Experiments have shown that a better classification effect can be obtained when the data arrangement direction is consistent with the direction of the convolution operation.

### 5.3. Model Comparison

**Figure 14:**
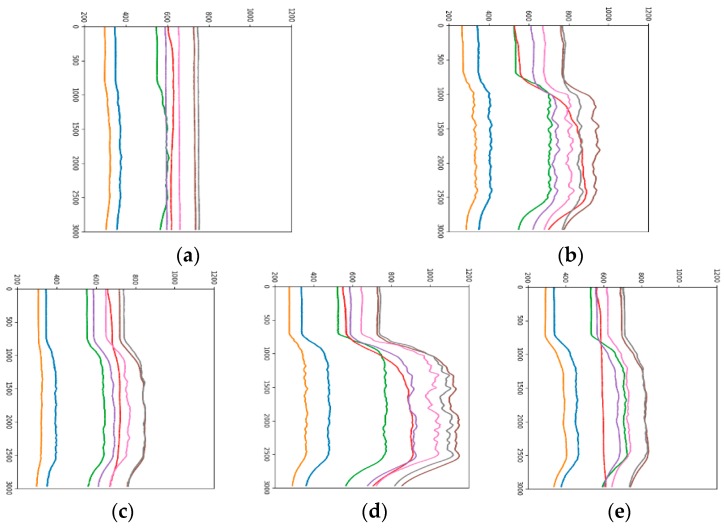
The samples of mixed gas in Sample-set 4 (**a**) Methane; (**b**) CO; (**c**) Ethylene; (**d**) CO-Ethylene; (**e**) Methane-Ethylene.

The samples of methane, CO, ethylene, CO-ethylene, methane-ethylene in Sample-set 4 are shown in Figure 14. Next, the test results and the performance of each network model are analyzed. Table 13 shows the recognition rates of the five mixed gases in Sample-set 4 by VGG-16, VGG-19, Resnet18, Resnet34 and Resnet50, as well as the average recognition rate of the algorithm and the average recognition rate of the categories.

The average recognition rate for each category from high to low is: methane > methane-ethylene > CO-ethylene > CO > ethylene. Specifically, methane has the highest recognition rate and the recognition rate of each model is 100%. The recognition rates of methane-ethylene and CO-ethylene are ranked second and third respectively. The recognition rate of the Resnet with high model complexity is 100%, while the recognition rate of VGG with simple network structure is slightly lower. The recognition rate of CO ranks fourth, and the recognition rate of VGG-16 network is the highest. It may be that the over-fitting phenomenon occurs when using a network with high complexity. The lowest recognition rate of ethylene is because the sample of this category consists of two parts, namely ethylene at a CO concentration of zero and ethylene at a methane concentration of zero. Each part contains three concentrations of ethylene, and the sensor responds differently to each concentration. In the case of a small sample size, accuracy will be greatly affected. 

The average recognition rate for each model from high to low is: Resnet50 > Resnet34 > Resnet18 > VGG-16 > VGG-19. Among them, Resnet50 has an average recognition rate of 93.334%, and Resnet50 has a classification accuracy of 100% for mixed gas except CO. The average recognition rate of VGG-19 is 71.112%, and the recognition rate of ethylene by VGG-19 is only 33.33%. Moreover, it is found that the recognition rate of Resnet is significantly higher than that of VGG. Because the Resnet model not only solves the gradient explosion problem that occurs when the VGG increases depth, but also extracts more comprehensive features of the gas data, thereby improving the accuracy.

**Figure 15:**
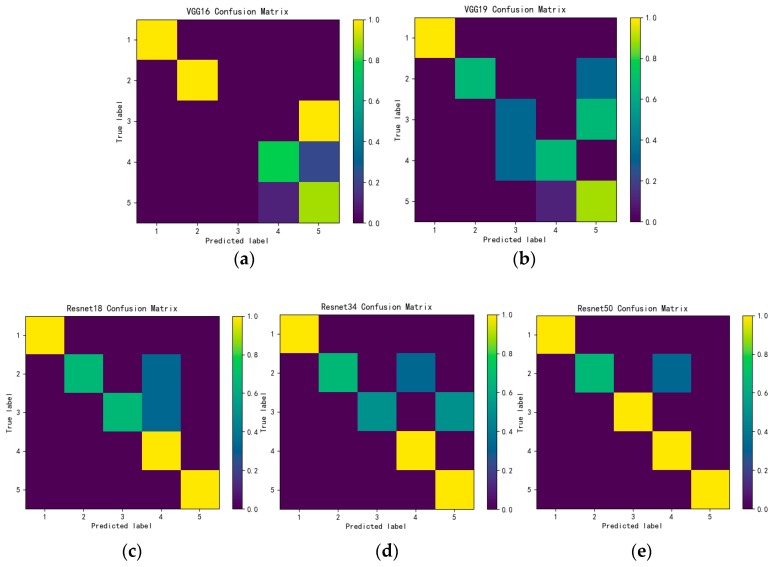
Confusion matrix results of five models (**a**) VGG-16; (**b**) VGG-19; (**c**) Resnet18; (**d**) Resnet34; (**e**) Resnet50.

To further evaluate the classification performance of each model, we use the confusion matrix to display the classification results. The confusion matrix is usually a specific matrix used to visualize the performance of the algorithm. The accuracy of the classification results of all categories can be displayed in a confusion matrix. As shown in Figure 15, since there are five categories, the confusion matrix is divided into 25 parts, and the color in each part represents the relative relationship between the real category and the predicted category. That is, the darker the color, the smaller the probability that the real category is predicted to be the corresponding predicted category. Therefore, by observing the color, the accuracy of each category can be intuitively perceived. That is, the lighter the color of the main diagonal portion means that the higher the accuracy and the better the prediction effect.

By analyzing the confusion matrix, VGG-16 completely recognizes ethylene gas as methane-ethylene. VGG-19 also classifies some ethylene samples as methane-ethylene, while Resnet 18 classifies some CO samples and ethylene samples as CO-ethylene. It shows that mixing methane or CO into ethylene has little effect on the sensor readings and the concentrations of gas in each category include low, medium and high. The concentration also affects the response of the sensor, which makes it difficult to identify the mixed gas. Resnet34 has improved the classification accuracy of ethylene. Resnet50 only recognizes part of CO as CO-ethylene, and the classification accuracy of other mixed gases reaches 100%, which directly proves that the classification performance of Resnet50 is optimal among the five models.

## 6. Conclusions

In this work, we propose a method for classifying mixed gas analogous-image matrix using convolutional neural networks. The proposed method applies the existing convolutional neural networks, which are mostly applied to visual picture classification, to the classification of gas time series data. This method provides a new research idea for mixed gas classification. Gas data is time series data which collected by the sensor that changes over timer. When considering multiple sequence values of the sensor array, it will constitute a two-dimensional matrix of m × n, and “m” is much larger than “n”. When extracting features directly from a matrix using convolutional neural network, “m” must be extracted, but this will inevitably lead to the loss of effective information, resulting in a decrease in accuracy. Therefore, this paper proposes to map the original two-dimensional time series matrix into a three-dimensional analogous-image matrix, mapping the original data values into pixel values in the analogous-image. And compare the impact of three mapping methods (fixed coordinate upper and lower limits, sensor baseline standardization and change data arrangement direction) on the classification results. 

Compared with the general pattern recognition algorithm, the proposed method does not have much requirements on the size and smoothness of the original data, and has good generalization. Moreover, it also works well in a big data environment. When there is a large amount of mixed gas data, there are more training samples correspondingly, so that more comprehensive features can be extracted, and the network accuracy will be improved. More importantly, the convolution operation in the proposed method can extract the deep features of the analogous-image and classify the mixed gas more accurately. Five kinds of convolutional neural networks of VGG-16, VGG-19, Resnet18, Resnet34 and Resnet50 were used for comparative experiments, the experimental results show that the Sample-set 4 with “fixed upper and lower limits of coordinate axes”, “sensor baseline non-standardization” and “vertical alignment of data” has the best classification results, which achieved a recognition rate of 96.67%. The performance of the network is further analyzed by the loss function and the Confusion matrix.

Our method has obtained good experimental results in the existing data sets and provides new ideas for the classification of mixed gas data. However, due to the insufficient number of samples, some categories with inconspicuous feature patterns are often confused with other categories, resulting in high error rates in some categories leading to a decrease in average accuracy. Therefore, in the future, we will further study how to improve the accuracy in the case of a small sample size.

## Figures and Tables

**Table 1:** The advantages and disadvantages of each method of identifying components in the gas mixture.

	Advantages	Disadvantages
GC-MS	Accurately identified	Expensive apparatus, time consuming, and specialized operator
Data-driven	Convenience and efficiency	Low accuracy for complex data
Fusion	Effectiveness and high accuracy	Process complicated and unstable result

**Table 2:** MOX sensors information in the sensor array.

Sensor Type	Number of Units	Channel	Target Gases
TGS2600	1	1	Hydrogen, carbon monoxide, methane
TGS2602	2	2,3	Ammonia, hydrogen sulfide, toluene
TGS2610	1	8	Propane, isobutane
TGS2611	1	7	Hydrogen, methane, isobutane
TGS2612	1	5	Methane, propane
TGS2620	2	4,6	Hydrogen, carbon monoxide, methane

**Table 3:** A sample of the original data.

Time(s)	TGS2600	TGS2602	TGS2602	TGS2620	TGS2612	TGS2620	TGS2611	TGS2610
3	331	273	518	572	566	700	629	719
3.1	331	274	518	571	566	700	630	719
3.2	333	273	518	571	566	701	630	719
⋯				⋯				
299.7	355	280	547	597	682	858	723	822
299.8	355	280	548	598	681	856	721	821
299.9	357	281	547	597	681	856	721	821

**Table 4:** Average concentration levels of three gases.

Concentration Level	CO (ppm)	Methane (ppm)	Ethylene (ppm)
zero	0	0	0
low	270	51	31
medium	397	115	46
high	460	131	96

**Table 5:** The standardized data.

Time	TGS2600	TGS2602	TGS2602	⋯	TGS2620	TGS2611	TGS2610
3	0	0	0		0	0	0
3.1	0	0.003663	0		0	0.00158983	0
3.2	0.0060423	0	0		0.00142857	0.00158983	0
3.3	0	0	0		0	0.00158983	0
⋯				⋯			
299.61	0.07250755	0.02564103	0.05598456		0.22571429	0.14785374	0.143255
299.7	0.07250755	0.02564103	0.05598456		0.22571429	0.14944356	0.143255
299.8	0.07250755	0.02564103	0.05791506		0.22285714	0.14626391	0.141864
299.9	0.078549849	0.029304029	0.055984556		0.222857143	0.146263911	0.1418637

**Table 6:** Performance evaluation environment.

Feature	Contents
Experimental system	Ubuntu16.04
CPU	Inter® Core (TM) i7 3.6GHz
GPU	NVIDIA Tesla p40 × 4
Memory	12GB × 2
Disk	2TB
Program language	Python

**Table 7:** Gas concentration configuration and number of samples for each category.

Category	Mixed Composition	Concentration Configuration	Total Number of Samples
1	Methane	(L, M, H)_Methane_ × (N)_Ethylene_	18
2	CO	(L, M, H)_CO_ × (N)_Ethylene_	18
3	Ethylene	(N)_CO_ × (L, M, H)_Ethylene_, (N)_Methane_ × (L, M, H)_Ethylene_	36
4	CO + ethylene	(L, M, H)_CO_ × (L, M, H)_Ethylene_	54
5	Methane + ethylene	(L, M, H)_Methane_ × (L, M, H)_Ethylene_	54

**Table 8:** Original dataset and extended dataset.

Category	Original Dataset		Extended Dataset	
	Training	Testing	Training	Testing
1	15	3	90	9
2	15	3	90	9
3	30	6	90	18
4	45	9	90	27
5	45	9	90	27

**Table 9:** Experimental results for different classification models.

Model	Model Parameter
VGG-16	epochs = 100, batch_size = 128, img_size = 224 × 224 × 3, lr = 0.001, pretrained = True
VGG-19	epochs = 100, batch_size = 128, img_size = 224 × 224 × 3, lr = 0.001, pretrained = True
Resnet18	epochs = 100, batch_size = 64, img_size = 640 × 480 × 3, lr = 0.001, pretrained = True
Resnet34	epochs = 100, batch_size = 64, img_size = 640 × 480 × 3, lr = 0.001, pretrained = True
Resnet50	epochs = 100, batch_size = 64, img_size = 640 × 480 × 3, lr = 0.001, pretrained = True

**Table 10:** Test accuracy for different classification models.

Model	Unfixed Range of Response Values (Sample-set 1)	Fixed Range of Response Values (Sample-set 2)
VGG-16	40%	63.33%
VGG-19	43.33%	60%
Resnet18	93.33%	90%
Resnet34	100%	93.33%
Resnet50	93.33%	96.67%
Mean	73.998%	80.67%

**Table 11:** Test accuracy for different classification models.

Model	Sensor Baseline Standardized (Sample-set 3)	Sensor Baseline non-Standardized (Sample-set 2)
VGG-16	56.67%	63.33%
VGG-19	46.67%	60%
Resnet18	90%	90%
Resnet34	90%	93.33%
Resnet50	96.67%	96.67%
Mean	76%	80.67%

**Table 12:** Test accuracy for different classification models.

Model	Horizontal Arrangement of Data (Sample-set 2)	Vertical Arrangement of Data (Sample-set 4)
VGG-16	63.33%	70%
VGG-19	60%	70%
Resnet18	90%	86.67%
Resnet34	93.33%	90%
Resnet50	96.67%	96.67%
Mean	80.67%	82.668%

**Table 13:** Identification results of VGG-16, VGG-19, Resnet18, Resnet50 and Resnet50.

Category	Mixed Gas	Detection Sample Recognition Rate/%					
		VGG-16	VGG-19	Resnet18	Resnet34	Resnet50	Mean
1	Methane	100	100	100	100	100	100
2	CO	100	66.67	66.67	66.67	66.67	73.336
3	Ethylene	0	33.33	50	66.67	100	50
4	CO-Ethylene	77.78	66.67	100	100	100	88.89
5	Methane-Ethylene	88.89	88.89	100	100	100	95.556
mean	—	73.334	71.112	83.334	86.668	93.334	—
